# Antimalarial activity of plumbagin *in vitro* and in animal models

**DOI:** 10.1186/1472-6882-14-15

**Published:** 2014-01-12

**Authors:** Wiriyaporn Sumsakul, Tullayakorn Plengsuriyakarn, Wanna Chaijaroenkul, Vithoon Viyanant, Juntra Karbwang, Kesara Na-Bangchang

**Affiliations:** 1 Chulabhorn International College of Medicine, Thammasat University (Rangsit Campus), Pathumtani 12121, Thailand; 2 Department of Clinical Product Development, Nagasaki Institute of Tropical Medicine, Nagasaki, Japan

**Keywords:** Plumbagin, Antimalarial, *Plasmodium falciparum*, *Plasmodium berghei*

## Abstract

**Background:**

Plumbagin is the major active constituent in several plants including *Plumbago indica* Linn. (root). This compound has been shown to exhibit a wide spectrum of biological and pharmacological activities. The present study aimed to evaluate the *in vitro* and *in vivo* antimalarial activity of plumbagin including its acute and subacute toxicity in mice.

**Methods:**

*In vitro* antimalarial activity of plumbagin against K1 and *3D7 Plasmodium falciparum* clones were assessed using SYBR Green I based assay. *In vivo* antimalarial activity was investigated in *Plasmodium berghei-*infected mouse model (a 4-day suppressive test).

**Results:**

Plumbagin exhibited promising antimalarial activity with *in vitro* IC_50_ (concentration that inhibits parasite growth to 50%) against 3D7 chloroquine-sensitive *P. falciparum* and K1 chloroquine-resistant *P. falciparum* clones of 580 (270–640) and 370 (270–490) nM, respectively. Toxicity testing indicated relatively low toxicity at the dose levels up to 100 (single oral dose) and 25 (daily doses for 14 days) mg/kg body weight for acute and subacute toxicity, respectively. Chloroquine exhibited the most potent antimalarial activity in mice infected with *P. berghei* ANKA strain with respect to its activity on the reduction of parasitaemia on day 4 and the prolongation of survival time.

**Conclusions:**

Plumbagin at the dose of 25 mg/kg body weight given for 4 days was safe and produced weak antimalarial activity. Chemical derivatization of the parent compound or preparation of modified formulation is required to improve its systemic bioavailability.

## Background

Malaria is widespread in tropical and subtropical regions. Throughout the history of mankind, this highly infectious disease has been one of the major causes of human illness and death. Chemotherapy with effective antimalarial drugs remains the mainstay for malaria control in the absence of a suitable vaccine treatment. *Plasmodium falciparum* is the most virulent and widespread infectious malarial species in tropical and subtropical countries due to the resistance of the parasite to most of the available antimalarial drugs [1]. In a race to combat the increasing multidrug *resistance P. falciparum*, artemisinin-based combination therapy (ACT) has been recommended by the World Health Organization (WHO) as the first-line treatment for acute uncomplicated multidrug resistance *P. falciparum* malaria in all the malaria endemic areas of the world. As antimalarial drug resistance compromises the effective treatment of the disease, there is a pressing need for ongoing drug discovery research that will provide effective, safe, and affordable antimalarial agents. Natural products including medicinal plants may offer relatively cheap alternative treatment opportunities for malaria patients [2,3]. Two antimalarial drugs currently widely used for malaria control originally came from indigenous medicinal plants; quinine is isolated from the Peruvian Cinchona's bark and artemisinins are obtained from the Chinese plant *Artemisia annua* Linn.

Plumbagin (5-hydroxy-2-methyl-1,4-naphthoquinone) is a natural product isolated from several plants in the families of *Plumbaginaceae, Droseraceae, Ancestrocladaceae,* and *Dioncophyllaceae.* It is a naphthoquinone that occurs in plant roots as a colorless combined form that can be processed to plumbagin by acid treatment [4]. This compound has been shown to display a wide spectrum of biological and pharmacological activities such as activities against malaria, leishmania and trypanosome parasites, as well as against virus, cancers, and insects [5]. The ethanolic extract of *Plumbago zeylanica* has been reported to exhibit *in vitro* antimalarial activity against chloroquine-sensitive clone of *P. falciparum* (3D7) with an IC_50_ (concentration that inhibits parasite growth by 50%) of 17 μg/ml [6]. The activity against *P. falciparum* enzyme succinate dehydrogenanse (SDH) including parasite growth has been shown to be inhibited to 50% by plumbagin at inhibitory concentrations of 5 and 0.27 mM, respectively [7]. Recently, our group has demonstrated promising antimalarial activity of the ethanolic extract of *Plumbago indica* Linn. [8]. The aim of the present study was to further evaluate the *in vitro* and *in vivo* antimalarial activity of its active constituent plumbagin. In addition, acute and subacute toxicity tests were performed to confirm its safety and tolerability, and to obtain an optimal dose used for the *in vivo* antimalarial evaluation.

## Methods

### Chemicals

Plumbagin (purity 98.2%) was obtained from Apin chemicals Co. Ltd. (Oxford, UK). Tween-80, and chloroquine diphosphate were obtained from Sigma-Aldrich (St. Louis, MO, USA). RPMI 1640 powder containing L-glutamine, streptomycin/penicillin, and HEPES were obtained from Gibco BRL Life Technologies (Grand Island, NY, USA). Gentamicin was obtained from Invitrogen Life Technologies Inc. (Carlsbad, CA, USA).

### *In vitro* experiment

#### In vitro cultivation of malaria parasite

Blood stages of the laboratory clones chloroquine-resistant (K1) and chloroquine-sensitive (3D7) *P. falciparum* were cultured *in vitro* according to the method of Trager and Jensens [9]. All culture steps were performed using aseptic technique in the NuAire laminar flow class II safety cabinet. All glassware was autoclaved at 121°C (15 atmosphere) for at least 15 min. Malaria parasite was maintained in continuous culture with human packed red blood cells (blood group O^+^) in RPMI 1640 medium supplemented with 10% human AB^+^ serum, 25 mM N-2-hydroxyethylpiperazine-N-2-ethanesulfonic acid (HEPES), 25 mM sodium bicarbonate, and gentamycin sulfate (60 μg/ml, pH 7.2). The culture was incubated at 37°C in an atmosphere consisting of 90% N_2_, 5% O_2_, and 5%. Parasite culture was synchronized to the ring stage by treatment with 5% (w/v) D-sorbitol [10].

#### Assessment of in vitro antimalarial activity of Plumbagin

Antimalarial activity of plumbagin was investigated using SYBR Green I assay [10,11]. Highly synchronous ring stage parasite was used in each assay. An aliquot of parasite inoculum (50 μl) with 2% parasitaemia and 1% haematocrit was added into each well of microtiter plate. Plumbagin (dissolved in DMSO and diluted with RPMI 1640 to final concentration of 1%) was added to the malaria culture at eight final concentrations of 210, 420, 840, 1680, 3360, 6720, 13440, and 26880 nM. Chloroquine (3.89-498.15 nM) and artesunate (0.39-50.0 nM) were used as standard antimalarial drugs.

The experiment was repeated three times (triplicate each). IC_50_ value (drug concentration that inhibits the parasite growth by 50%) was used as an indicator of antimalarial activity and was determined from a log-dose–response curve plotted using the Calcusyn™ version 1.1 (BioSoft, Cambridge, UK).

### *In vivo* experiments

#### Animals

ICR (Imprinting Control Region) mice (5–7 weeks of age, weighting 20–40 g) of both sexes were used in the study. All were obtained from the National Laboratory Animal Centre, Thailand. Animal experiments were carried out in accordance with the OECD Guideline for Chemicals [12]. The animals were housed under standard conditions and fed with a stock diet and water *ad labitum*. Approval of the study protocol was obtained from the Ethics Committee for Animal Research, Thammasat University, Thailand.

#### Toxicity tests

Plumbagin was weighted and resuspended with 20% Tween-80 to obtain the desired concentrations. ICR mice were fasting 2 h before feeding with a single oral dose of plumbagin. Animals were divided into eight groups of six (3 males and 3 females for each group). For the acute toxicity test, mice in each group were fed with plumbagin at a single oral dose of 500, 200, and 100 mg/kg body weight; control group received a single oral dose of 20% Tween-80 (1 ml). For the subacute toxicity test, mice in each group were fed with plumbagin at a daily oral dose of 100, 50, and 25 mg/kg body weight for 14 days; control group received a daily oral dose 1 ml of 20% Tween-80 for 14 days [13,14]. General behavior of each mouse was observed continuously for 1 h after each dose, intermittently every 4 h, and thereafter over a period of 24 h [15]. Animals were observed for up to 14 days for the acute toxicity test and 28 days for the subacute toxicity test for any sign of toxicity (behavioral change related to central nervous, cardiovascular and gastrointestinal systems as well as complete blood count, liver and kidney function tests), body weight change and water and food consumption. At the end of the observational period, all animals were sacrificed under ether anesthesia and vital organs (heart, lung, liver, spleen and kidney) were removed from all animals for gross and histopathological examination.

#### Assessment of antimalarial activity of plumbagin in Plasmodium berghei-infected mouse model (4-day suppressive test)

The *in vivo* antimalarial activity of plumbagin was evaluated using a 4-day suppressive test in *P. berghei*-infected mouse model [16]. *P. berghei* (ANKA) strain used in the experiment was obtained from the National Center for Genetic Engineering and Biotechnology (BIOTEC), Thailand. The parasite had been maintained by serial blood passage in mice, and blood stage stored at -196°C until use.

ICR mice were divided into five groups (3 males and 3 females for each group). The donor mice were infected with 200 μl of *P. berghei* parasite inoculum. The parasitized blood of each donor mouse was collected from the tail vein and diluted with 0.9% sodium chloride. Mice were infected with saline suspension of 1 × 10^7^ parasitized erythrocytes (0.2 ml) by intraperitoneal injection (Day 0). Four hours after infection, animals were treated with plumbagin at oral daily doses of 1, 10, or 25 mg/kg body weight of plumbagin for four consecutive days (test group 1, 2 and 3, respectively). Positive and negative control groups were fed with the antimalarial chloroquine at oral daily doses of 10 mg/kg body weight of plumbagin and 20% Tween-80, respectively. On day 4 (96 hours post infection), parasitaemia of individual mouse was determined under light microscope by examination of giemsa-stained thin blood smears prepared from mouse tail blood [17]. The mean parasitaemia in each group of mice was used to calculate the % suppression for each dose using the formula:

$$\%\text{Suppression}=\frac{\text{Parasitaemia of negative control}-\text{Parasitaemia of test}}{\text{Parasitaemia of negative control}}\times 100$$

The antimalarial activity of plumbagin was determined from the ratio of percentage of parasite reduction in treated and negative control groups [18]. Results are expressed as median (range) values. Comparison of difference in quantitative variables between more than two and two groups was performed using Kruskal Wallis and Mann–Whitney U tests (SPSS version 16.0, SPSS Inc., CO, USA). Statistical significance level was set at α < 0.05 for all tests.

## Results

### Assessment of *in vitro* antimalarial activities of Plumbagin

The median IC_50_ values for antimalarial activity of plumbagin against 3D7 chloroquine-sensitive *P. falciparum* and K1 chloroquine-resistant *P. falciparum* clones were 580 and 370 nM, respectively. The corresponding IC_50_ values for chloroquine and artesunate were 10.5 *vs* 128.7 and 2.1 *vs* 1.91 nM, respectively (Table 1).

**Table 1:** ***In vitro *** **antimalarial activity of plumbagin, chloroquine and artesunate**

**Compound**	**IC**_**50 **_**against 3D7 chloroquine-sensitive *P. falciparum *** **clones (nM)**	**IC**_**50 **_**against K1 chloroquine resistant *P. falciparum *** **clones (nM)**
Plumbagin	580 (270–640)	370 (270–490)
Chloroquine	10.5 (9.4–12.1)	128.7 (109.3–139.2)
Artesunate	2.1 (1.98–2.54)	1.91 (16.1–2.1)

Data are presented as median (range) of IC_50_ values (nM).

### Toxicity tests

**Figure 1:**
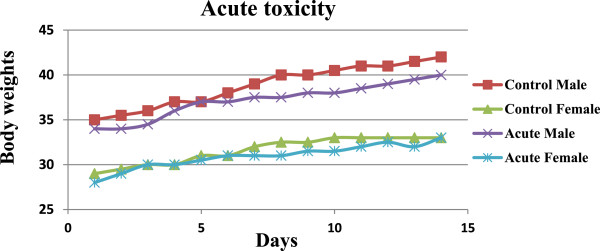
Median body weight (g) of male and female mice (n = 6 for each group) during the first 14 days in the administration of a single oral dose of 100 mg/kg body weight of plumbagin and following Tween-80 (control) in the acute toxicity test.

The toxicity of plumbagin when given as a single oral dose (acute toxicity) and 14-day daily doses (subacute toxicity) in mice was investigated in order to define optimal dose of plumbagin to be used for evaluation of its *in vivo* antimalarial activity in malarial mouse model. Results indicated virtually no toxicity of plumbagin at a maximum single oral dose of 100 mg/kg body weight (acute toxicity). All mice survived following a single oral dose of 100 mg/kg body weight of plumbagin and 20% Tween-80 (control) (Table 2). There was neither sign of toxicity nor significant change in water and food consumption and body weights of mice in both groups during the 14 days observation period (Figure 1). Toxic signs and symptoms including anxiety and agitation were however observed in 6/6 and 2/6 of mice following the doses of 500 and 200 mg/kg body weight, respectively; all subsequently died within 24 hours. The gross examination of vital organs, *i.e.,* heart, lung, liver, spleen and kidney in both treated (all dose levels) and control groups were similar either in size and cell morphology.

**Table 2:** Number of survived ICR mice following a single (acute toxicity) and multiple (subacute toxicity) oral dosing of plumbagin

	**Test group**	**Number of mice (survived/total)**
**Acute toxicity**	Control (20% Tween-80)	6/6
	Plumbagin: 500 mg/kg body weight	0/6
	Plumbagin: 200 mg/kg body weight	4/6
	Plumbagin: 100 mg/kg body weight	6/6
**Subacute toxicity**	Control (Tween-80)	6/6
	Plumbagin: 100 mg/kg body weight	0/6
	Plumbagin: 50 mg/kg body weight	0/6
	Plumbagin: 25 mg/kg body weight	6/6

**Figure 2:**
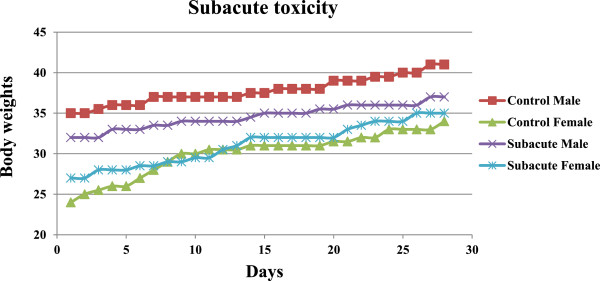
Median body weight (g) of male and female mice (n = 6 for each group) during the 28 days following the administration of daily oral doses of 25 mg/kg body weight of plumbagin and Tween-80 (control) in the subacute toxicity test.

For the subacute toxicity test, all mice survived following daily oral doses of 25 mg/kg body weight plumbagin and 20% Tween-80 (control) for 14 days (Table 3). There was neither abnormality in behavior, sign of toxicity, nor significant change in water and food consumption and body weights during the 14 days observation period (Figure 2). Toxic signs and symptoms including anxiety and agitation were however observed in all mice following the doses of 100 and 50mg/kg body weight of plumbagin (for 14 days). Mice receiving 100 mg/kg body weight of plumbagin died within 4–8 days, whereas those receiving 50 mg/kg body weight dosing died within 8–11 days. The gross examination of vital organs, *i.e.,* heart, lung, liver, spleen and kidney in both treated and control groups were similar in size and cell morphology.

**Table 3:** **Antimalarial activity of plumbagin compared with chloroquine and negative control (treated with 20% Tween-80) against *P. berghei *** **ANKA strain in mice (4-day suppressive test)**

**Treatment**	**Dose (mg/kg body weight/day)**	**Parasite density (range: %) on day 4**	**% Suppression at day 4**	**Survival time (days)**
**Negative control (20% Tween 80)**	-	37.8 (46.9-41.8)^a^	0	7 (6–7)
**Plumbagin**	1	35.6 (31.7–39.6)	5.8	7.5 (6–9)
	10	35.05 (31.2–38.8)	7.3	8 (8–9)
	25	22.3 (19.2–25.7)	41	10 (9–11)
**Chloroquine**	10	0 (0–0)^b^	100^c^	> 15^d^

Data are presented as median (range) values (6 mice for each group) of parasite density, % suppression and survival time.

^*a*^*Significantly lower than the groups treated with 10 and 25 mg/kg body weight/day of plumbagin and 10 mg/kg body weight/day of chloroquine (p < 0.05).*

^*b*^*Significantly higher than negative control and the groups treated with 1, 10 and 25 mg/kg body weight/day of plumbagin (p < 0.05).*

^*c*^*Significantly higher than negative control and the groups treated with 1, 10 and 25 mg/kg body weight/day of plumbagin (p < 0.05).*

^*d*^*Significantly longer than the groups treated with 1, 10 and 25 mg/kg body weight/day of plumbagin and 10 mg/kg body weight/day chloroquine (p < 0.05).*

### Assessment of antimalarial activity of plumbagin in *Plasmodium berghei*-infected mouse model (4-day suppressive test)

Results of the 4-day suppressive antimalarial test of plumbagin and chloroquine in mice infected with *P. berghei* ANKA strain are summarized in Table 3. Median (range) parasite density on day 4 of the negative control group (20% Tween-80), mice treated with 1, 10 and 25 mg/kg body weight of plumbagin, and 10 mg/kg body weight of chloroquine for 14 days were 37.8 (46.9-41.8), 35.6 (31.7-39.6), 35.05 (31.2-38.8), 22.3 (19.2-25.7) and 0 (0–0)% respectively. Parasite density on day 4 in the control group treated with Tween-80 was higher than the groups treated with chloroquine and 25 mg/kg body weight plumbagin. Chloroquine exhibited the most potent antimalarial activity with respect to its activity on reduction of parasitaemia on day 4 and prolongation of survival time. Parasite density (%) on day 4 following chloroquine treatment (0%) was significantly lower than 20% Tween 80 (negative control) and plumbagin at all dose levels (*p* < 0.05). In addition, parasite suppression (%) of mice treated with chloroquine (100%) was significantly higher than the negative control group (0%) and the groups treated with 1 (5.5%), 10 (7.3%), and 25 (41%) mg/kg/day plumbagin (*p* < 0.01). The survival time in the group treated with chloroquine was also significantly longer than the negative control and the groups treated with plumbagin at all dose levels (*p* < 0.01).

## Discussion

Plumbagin, a naturally occurring naphthoquinone widely distributed in the *Plumbaginaceae* family, has been reported to possess a wide spectrum of pharmacological properties. The crude ethanolic extract of *Plumbago indica* Linn. (root) has been shown to possess good to moderate antimalarial activity (class III antimalarial activity) in our previous *in vitro* screening [8]. Among the 32 plants investigated, *Plumbago indica* Linn. showed the most promising activity against both K1 chloroquine-resistant (IC_50_ 3 μg/ml) and 3D7 chloroquine-sensitive (IC_50_ 6.2 μg/ml) clones, with highest selectivity (SI = 44.7 and 21.6, respectively). Its antimalarial potency against K1 *P. falciparum* clone was about 2.2% of artesunate. In the present study, promising antimalarial activity of its active constituent plumbagin was initially demonstrated in the *in vitro* assay with median IC_50_ values against 3D7 chloroquine-sensitive and chloroquine-resistant *P. falciparum* clones of 580 and 370 nM, respectively. It was noted for a relatively higher activity of plumbagin against chloroquine-resistant *P. falciparum* (class I: very good activity) compared with chloroquine-sensitive *P. falciparum* (class II: good) clone [19]. The difference in antimalarial activity between the two clones could be due to the difference in drug transportation mechanisms particularly those involving *Plasmodium falciparum* chloroquine resistance transporter (*pfcrt*). Parasite chromosomal loci associated with these differential chemical phenotypes should be investigated to clarify this issue [20]. This activity however, should be of advantage for the treatment of patients in areas where *P. falciparum* is still sensitive to chloroquine. Promising antimalarial activity was observed in the absence of significant toxicity both in acute and subacute toxicity testing with dose up to 100 mg/kg body weight and 25 mg/kg body weight/day for 14 days, respectively.

Based on the results of the *in vivo* antimalarial testing, plumbagin at the dose of 25 mg/kg body weight given for 4 days exhibited moderate to weak antimalarial activity with regards to its inhibitory activity on the reduction of parasitaemia and the prolongation of survival time. The compound at daily doses of 10 mg/kg body weight/day for 4 days showed only weak activity, while at daily doses of 1 mg/kg body weight did not produce any significant activity [19]. The antimalarial drug chloroquine exhibited the most potent antimalarial activity with 100% suppression of parasitaemia on day 4 (0% parasite density) and a significant prolongation of survival time (> 15 days). This result of the *in vivo* antimalarial activity of plumbagin was however, inconsistent with the observed *in vitro* showing the compound to be of good to moderate antimalarial activity. Plumbagin is poorly water soluble which results in poor absorption across gastrointestinal mucosa and thus low systemic bioavailability [21]. In a previous study, mice treated with liposomal formulation of plumbagin was shown to achieve higher plasma and tissue level and area under the concentration-time curve (AUC) compared with those treated with the water-soluble plumbagin. Moreover, high concentration was found in liver and spleen of mice [22]. *In vivo* pharmacokinetics study also demonstrated that orally administered plumbagin produced only 39% systemic bioavailability due to its limited biopharmaceutical properties such as high lipophilicity (log P 3.04) and insolubility in water [23].

## Conclusions

Plumbagin at the dose of 25 mg/kg body weight given for 4 days was safe and produced weak antimalarial activity. Chemical derivatization of the parent compound or preparation of modified formulation is required to improve its systemic bioavailability.

## Competing interests

The authors declare that they have no competing interest.

## Authors’ contributions

WS carried out all the *in vivo* experiments and drafted the manuscript. TP performed the toxicity tests. WC contributed to the *in vitro* experiments. VV and KJ contributed to the design of the study. KN contributed to revising the manuscript. All authors have read and approved the final version of the manuscript.

## Pre-publication history

The pre-publication history for this paper can be accessed here:

http://www.biomedcentral.com/1472-6882/14/15/prepub
